# Remission of severe aphthous stomatitis of celiac disease with etanercept

**DOI:** 10.1186/1476-7961-11-6

**Published:** 2013-12-24

**Authors:** Adey Hasan, Hiren Patel, Hana Saleh, George Youngberg, John Litchfield, Guha Krishnaswamy

**Affiliations:** 1 The Department of Internal Medicine, East Tennessee State University, Johnson City, TN, USA; 2 Division of Allergy, Asthma and Immunology, East Tennessee State University, PO Box 70622, Johnson City, TN, USA; 3 James H. Quillen VA Medical Center, East Tennessee State University, Johnson City, TN, USA; 4 Department of Pathology, Quillen College of Medicine, East Tennessee State University, Johnson City, TN, USA; 5 Department of Medicine, Quillen College of Medicine, Johnson City, TN 37614-0622, USA

**Keywords:** Celiac disease, Gluten-sensitivity, Aphthous stomatitis, Etanercept, Tumor necrosis factor

## Abstract

Celiac disease is a common autoimmune disease triggered by gluten-containing foods (wheat, barley and rye) in genetically predisposed individuals. We present a patient with celiac disease complicated by severe aphthous stomatitis resulting in impairing swallowing, chewing and speaking. This led to weight loss, psychosocial problems as well as inability to perform her work. A variety of topical and systemic medications used resulted in either no improvement or only partial alleviation of the patient’s symptoms. After informed consent, etanercept was initiated and resulted in complete remission of aphthous stomatitis, decrease in arthralgia and fatigue and considerable improvement in her quality of life. The use of newer biological agents for selected and severe manifestations of celiac disease may lead to improved morbidity in these patients, but more studies are needed to determine long-term efficacy as well as safety of these drugs in the mucosal and/or systemic complications of this disease.

## Introduction

Celiac disease (CD) is a common autoimmune disease. The prevalence of CD is approximately 0.5% to 1% among people living in the United States [1] and 1:100 to 1:300 worldwide [2]. It is more common in Caucasians and affects both children and adults, with a female predominance [1]. CD- otherwise known as Gluten-sensitive enteropathy- is triggered by gluten-containing foods (wheat, barley and rye) in genetically predisposed individuals and can also be associated with other immunological diseases such as diabetes mellitus type 1 and IgA deficiency, suggesting immune dysregulation. Although patients are often asymptomatic, CD can manifest with cutaneous, mucosal, systemic or autoimmune features [3,4]. A variety of oral lesions such as atrophic glossitis and aphthous ulcers are quite common in patients with CD with A prevalence ranging from 3% to 61% in several studies. Aphthous stomatitis can be quite severe in CD, at time interfering with chewing, swallowing, speaking, and leading to impaired quality of life. This can result in complications such as weight loss, nutrition deficiencies, depression and psychosocial withdrawal.

We present a patient with severe aphthous stomatitis complicating CD who responded partially to immune suppression, but dramatically to the inhibitor of tumor necrosis factor alpha (TNFα) - Etanercept. This report suggests a role for cytokines such as TNFα in CD, and also provides a potential treatment strategy for selected mucosal complications associated with the disease.

## Case report

A 32 year old Caucasian female with a history of endometriosis and fibrocystic breast disease presented with severe ulcers of the mouth extending into the posterior oropharynx. The presence of the ulcers was associated with severe pain with difficulty eating, chewing or swallowing, and interfered with her speech and quality of life. The lesions appeared initially as blisters and were followed by ulceration. These appeared in clusters over weeks or months leading to severe disability. Periodically, the patient would develop conjunctival injection, arthralgias and severe fatigue associated with the ulcers. A thorough ophthalmological evaluation was negative for glaucoma, cataracts, uveitis or scleritis.

**Figure 1:**
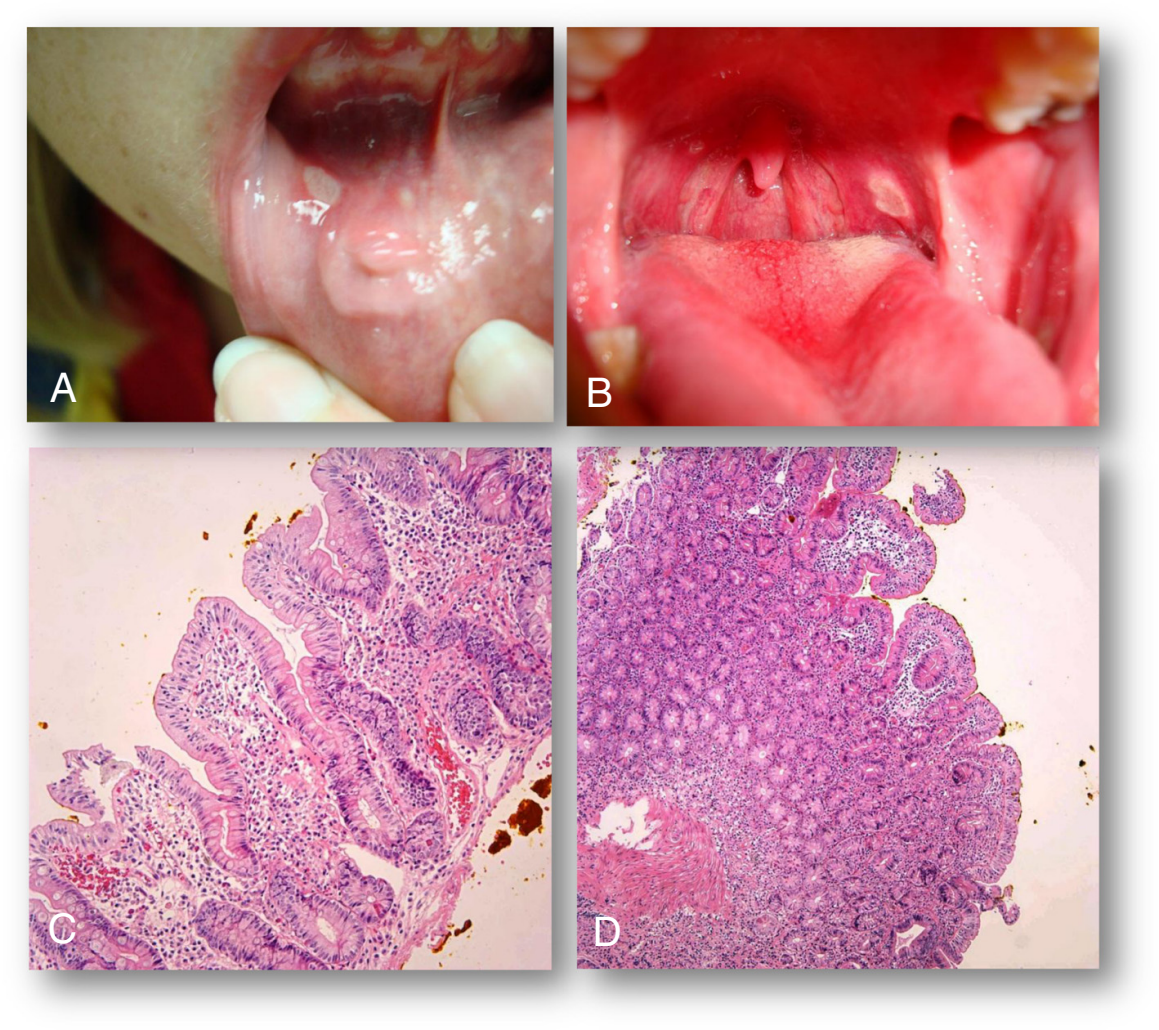
**Images of physical examination findings and biopsy results. A-D**: Gross examination of the oral mucosa demonstrated multiple aphthous ulcers and erythema. **(A)** A large, painful white ulcer with surrounding erythema located on the soft palate is present. **(B)** Demonstrates large, shaggy, inflamed ulceration of the lower lip. Granulation tissue, scarring and ulceration were also observed on biopsy of the lower labial mucosa. **(C & D)** Ileal biopsy revealed villous blunting, crypt elongation, increased inflammation in the lamina propria, and increased intraepithelial lymphocytes (**C**-low power, **D**-high power).

The past medical history included allergic rhinitis complicated by recurrent sinusitis. She also complained of tingling, pain and erythema with blanching of her fingers, consistent with Raynaud’s phenomenon. The patient’s medications included fexofenadine, montelukast sodium, intranasal steroids, tryptan for migraine and sertraline. A recent gynecological examination was unremarkable. Her family history was significant for a first cousin with systemic lupus erythematosus (SLE). Physical examination revealed multiple large aphthous ulcers with involvement of the buccal mucosae, tongue and palate (Figure 1A and B). A few lymph nodes in the upper neck were slightly enlarged. Raynaud’s phenomenon and livedoid changes were seen in the upper extremities.

A thorough evaluation demonstrated normal liver function tests, low levels of Vitamin D, ferritin (19 ng/ml) and thiamine (52 ng/ml) with normal levels of B12 and red blood cell folate (Table 1). Tests for autoantibodies and anti-cardiolipin antibodies were negative. Evaluation for CD demonstrated significantly elevated levels of endomysial and tissue transglutaminase (TTG) antibodies (Table 2). Biopsies of the esophagus demonstrated typical reflux changes, while biopsies of the ileum showed villous blunting, mononuclear and T cell infiltration of the lamina propria and epithelial loss (Figure 1C and D), confirming the diagnosis of CD. Genetic evaluation and HLA-DQ typing demonstrated the presence of celiac disease permissive alleles DQ alpha 1 0103, 0501 and DQ beta 1 0201, 0603, further clinching the diagnosis. These results are summarized in (Table 2), colonoscopy and biopsy were negative for Inflammatory bowel disease. Biopsy of oral lesion did not reveal vasculitis or Behcets syndrome.

**Table 1:** Other laboratory test results on patient

**Name**	**Result**	**Normal value**	**Comments**
**Anti-nuclear Ab (U/ml)**	46	0-100	Normal
**Anti-SSA Ab (U/ml)**	44	0-100	Normal
**Anti-SSB Ab (U/ml)**	7	0-100	Normal
**Cardiolipin IgG (GPL)**	7	<15	Normal
**Cardiolipin IgM (MPL)**	8	<12	Normal
**Cardiolipin Ab IgA (APL)**	2	<12	Normal
**Rheumatoid factor (IU/ml)**	<5	0-10	Normal
**Anti-CCP Ab (Units)**	3	<20	Normal
**C3 complement (mg/dL)**	119	89-187	Normal
**C4 complement (mg/dL)**	46.3	16.5-38.0	Increased-acute phase
**Ig A (mg/dL)**	82	66-436	Normal
**Vitamin B1 (nmol/L)**	52	70-180	Low
**Ferritin (ng/mL)**	19	6-159	Fe deficiency
**Vitamin B12 (pg/mL)**	425	193-982	Normal
**RBC folate (ng/mL)**	486	280-903	Normal
**TSH (IU/ml)**	2.28	0.4-4.0	Normal
**WBC (K/microL)**	4.4	4.8-10.8	Mild leukopenia
**Hgb (g/dL)**	11.4	12.0-16.0	Decreased; anemia

GPL = IgG phospholipid units; MPL = IgM phospholipid units; APL = IgA phospholipid units; CCP = cyclic citrullinated peptide; pg/Ml = Picograms/milliliter; RBC = Red Blood Cell; TSH = Thyroid Stimulating Hormone; WBC = White Blood Cell; Hgb = Hemoglobin.

**Table 2:** Diagnostic immunological test results

**Test**	**Result**	**Normal value**	**Comments**
**Gliadin IgG Ab (Units)**	69	<19 negative; 19–31 weak positive; >31 positive	Positive suggestive of celiac disease
**Gliadin IgA Ab (Units)**	11	<19	Normal
**Transglutaminase IgA Ab (Units)**	72	0-19	Increased suggestive of celiac disease
**Tissue transglutaminase IgG antibody (Units)**	5	<20	Normal
**Endomysial antibody titer**	1:40	<1:10	Increased suggestive if celiac disease
**DQ alpha 1 (MHR)**	0103,0501		Positive; indicating genetic predisposition
**DQ beta 1 (MHR)**	0201,0603		Positive; indicates genetic predisposition

IgG Ab = Immunoglobulin G Antibodies; IgA Ab = Immunoglobulin A Antibodies; MHR = Medium-High Resolution.

Tetracycline swish and swallow as well as topical lidocaine for the ulcers were initially prescribed. Replacement with Vitamin D, thiamine and iron were initiated and the patient was instructed to follow a strict gluten-free diet. Reflux symptoms were also treated with a proton pump inhibitor. The oral ulcers showed no response to tetracycline and lidocaine but a partial response to systemic glucocorticoids was seen, with relapse of symptoms soon after discontinuation. A repeat endoscopy showed normalization of the villous architecture in response to treatment; however, the aphthous ulcers persisted unabated. The patient continued to require more prednisone and narcotics for pain. A lip biopsy was performed and demonstrated mucositis and granulation tissue. Fungal stains and PCR for herpes simplex virus were negative.

The patient was started on and failed hydroxychloroquine, sucralfate, cyclosporine, azathioprine and colchicine. After informed consent and insurance approval, the patient was started on etanercept at a dose of 25 mg biweekly administered subcutaneously, after an initial negative tuberculin skin test. After adequate instruction, the patient was able to comfortably self-administer the medication. Pulmicort® Respules were prescribed for minor flares. After Etanercept was initiated, the patient had complete remission of the oral ulcers. She has also had dramatic improvements in the inflammatory symptoms including conjunctival inflammation, polyarthralgia and fatigue.

## Discussion

### Celiac disease and its manifestation

**Figure 2:**
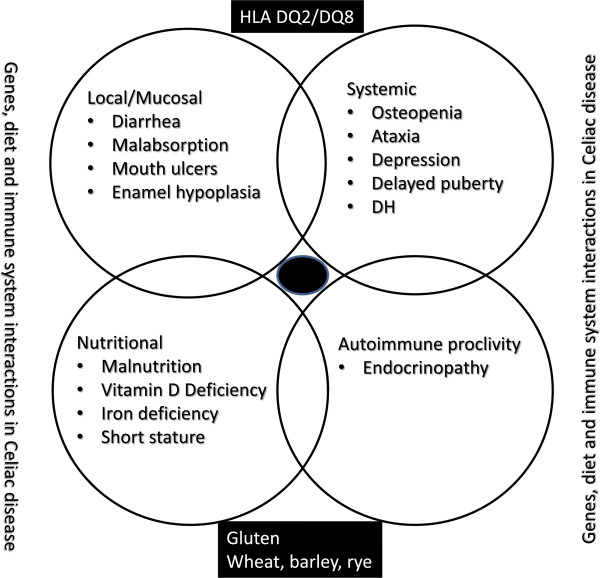
Celiac disease and its complications/associations.

Celiac disease is a chronic inflammatory, autoimmune disorder of the small intestine with local and systemic manifestations (Figure 2) [5-7]. The latter include mucosal disease (dental/enamel hypoplasia, malabsorption syndrome, aphthous stomatitis), dermatitis herpetiformis, osteopenia/osteoporosis, nutritional issues such as short stature and malnutrition with hypovitaminoses, infertility and delayed puberty, seizures and/or ataxia, depression and autoimmune endocrinopathy [5-7].

**Figure 3:**
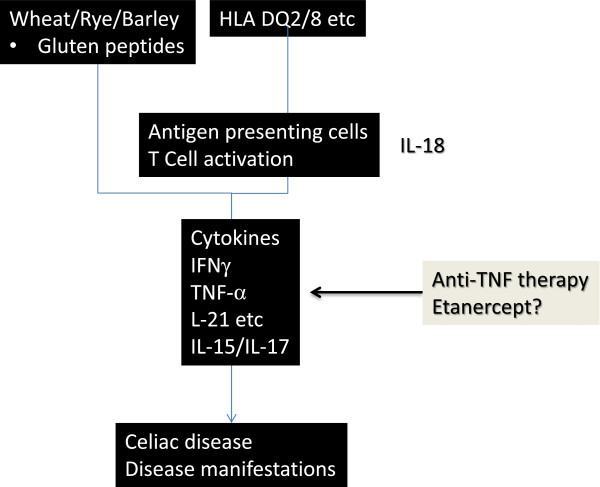
Immunopathogenesis of celiac disease and possible point of action of etanercept.

Pathogenesis of CD is an interaction between immunological factors and environmental factors in genetically susceptible population, through activation of both cell-mediated and humoral immune mechanisms [3,5-7]. After ingestion of dietary sources of gluten (wheat, barley and rye), gluten peptides, modified deamidated by tissue transglutaminases expressed in the lamina propria of the small intestines, are presented by professional antigen presenting cells to gluten-specific CD4 T cells in lamina propria of the intestinal cells and oral mucosa, in the context of class II HLA molecules (DQ2 and 8). Gliadin-reactive T cells in turn likely express interferon gamma (IFNγ), a T helper type 1 cytokine, which in turn activates metalloproteinases, resulting in some manifestations of the disease. It is also likely that IFNγ enhances the production of TNFα which may play a major role in recruitment of lymphocytes and mucosal damage [8]. Possible sequence of events and the point of action of Etanercept are shown in cartoon format in (Figure 3).

Of relevance to the patient discussed in this report, Recurrent Aphthous Ulcers (RAU) have been reported in patients with celiac disease, with variable frequencies [9-11]. Some studies have shown no higher frequency of RAU in CD compared to controls while others have demonstrated more frequent occurrence. Cell mediated immunity and/or formation of immune complexes may play a part in RAU development in patients with CD, though the actual mechanisms are unclear. Studies from peripheral blood in otherwise healthy patients with RAU, showed multiple immune system abnormalities. These abnormalities included: depressed or reversed CD4:CD8 cell ratio (especially in patients demonstrating severe aphthous stomatitis), increased γδ receptor-positive T cells in patients with active RAU compared to controls, and increased TNFα production [12]. In one study, patients with RAU had elevated salivary levels of TNFα compared to controls [13]. Increased numbers of γδ + cells suggest an antibody-dependent cell-mediated cytotoxicity in RAU pathogenesis. These specific T cells probably produce TNF-α, a major mediator responsible for the initiation of the inflammatory response through its effects on endothelial cell adhesion and neutrophil chemotaxis. Other inflammatory changes found in RAU lesions include elevated IL-2 and IL-6, potentially due to local tissue trauma. IL-2 can activate natural killer (NK) cells. Increased activity of these cells has been seen in active lesions, which also seems to diminish during remission. These inflammatory mediators may also have been responsible for the nonspecific symptoms suffered by the patient.

## Response of aphthous stomatitis to diet and biological response modifiers

Based on the immunological changes observed in RAU, biological response modifiers have been attempted by various physicians and researchers in an attempt to ameliorate or induce remission in severe disease. Systemic medications reported in the literature include pentoxifylline [14], doxycyline [15], Zinc Sulfate [16], Colchicine [17], clofazimine [18], prednisone [19], montelukast sodium [19], sulfones [20], thalidomide [21], methotrexate [22], cyclosporin, chlorambucil [23], infliximab [24], etanercept [25], interferon α [26], azathioprine [25] and levamisole [27] (Table 3).

**Table 3:** Systemic medications which have been used for treatment of RAU

**Medication**	**Number of patients**	**Results/outcome**	**Reference**
Pentoxifylline 400 mg TID	26 people	↓ ulcer size	[14]
	14 patients	↓ ulcers number	
	12 control		
Doxycyline 20 mg BID	50 patients	↑ remission	[15]
		↓ pain	
Zinc sulfate 150 mg BID	45 patients	↓ ulcer size	[16]
		↓ symptoms	
Cholchicine (1–2) mg BID	169 patients	↓ ulcer number	[17]
		↓ disease duration	
Clofazimine 100 mg QD For 1 month	23 patients	↓ ulcers number	[18]
		↓ disease duration	
		In 44%	
Prednisone up to 25 mg	60 patients	↓ ulcers number	[19]
		Faster healing	
Montelukast 10 mg QD	60 patients	↓ ulcers number	[20]
		Faster healing	
Thalidomide 50 mg QD 200 mg QD for HIV patients	20 HIV patients	Induce remission	[21]
		In HIV patients	
Chlorambucil 0.1 mg/kg	Single case report	Remission	[22]
Infliximab 5 mg/kg QD	Single case report	Remission	[23]
Etanercept 25 mg BiWeekly	40 patients	↓ ulcers number	[24]
Levamisole	9 patients	↓ ulcers number	[25]

Recent advances in understanding of molecular inflammatory mechanisms have led to new treatments for inflammatory disease- including tumour necrosis factor-alpha antagonists, infliximab, etanercept and adalimumab and the T-cell modulator modifiers efalizumab and alefacept [28]. Since recent studies have demonstrated a role for the newer TNFα inhibitors in RAS [29-31], this intervention was initiated in the patient, after informed consent.

In small case clusters or isolated reports, inhibition of TNFα has been shown to alleviate aphthous stomatitis. In one study, Infliximab, a chimeric anti-TNFα antibody, administered intravenously was quite effective in managing recurrent or refractory oral and genital ulcers, with no evidence of recurrence [24]. In another study, Etanercept, a fusion protein of the TNF-α receptor and the Fc portion of human IgG1, had a favorable effect on RAS of the oral cavity. In one review, sixteen patients underwent treatment for refractory or severe aphthous ulcers with tumor necrosis factor alpha antagonists, between 1995 and 2010 [32]. Of the drugs used, infliximab, etanercept and adalimumab appeared to improve outcomes [32]. Sanchez-Cano et al., also reported improved outcomes with adalimumab in a patients with severe aphthous ulcers [33]. The role of cytokines such as TNFα in severe aphthous stomatitis, especially in underlying disorders such as CD needs to be clarified.

Approved uses for etanercept include treatment of rheumatoid arthritis, juvenile rheumatoid arthritis, psoriatic arthritis, plaque psoriasis and ankylosing spondylitis. Recently this drug has also been utilized to treat complications in patients with HIV infection [34]. Off-label use has extended to include Behcet’s disease, recurrent aphthous stomatitis, pemphigoid and lichen planus [29]. A study done by Pepple K. et al., showed that etanercept was used for treatment of Alzheimer disease with small significant improvement [35]. Another study done by Zalevsky et al., showed that the use of etanercept in HIV/AIDS does not increase morbidity or mortality; in contrast it may improve associated aphthous stomatitis, fatigue and dementia [36]. As with any other immunosuppressant, etanercept increases the risk of infections, lupus, systemic sclerosis and congestive heart failure. Etanercept may also increase risk of lymphoma, central nervous system disease such as multiple sclerosis, vasculitis and autoimmune hepatitis but in clinical trials appears to have a favorable risk-benefit ratio [37-39]. In the patient mentioned in this report, dramatic improvement in mucosal disease as well as in the systemic complications of fatigue an arthralgia was convincing enough to allow her to stay on etanercept, though the long term safety and efficacy are unknown and need to be evaluated before firm recommendations on such use could be recommended.

## Conclusion

This patient had severe aphthous stomatitis complicating CD, impairing swallowing, chewing and speaking. This led to weight loss, psychosocial issues as well as inability to perform her work. A variety of topical and systemic medications used resulted in either no improvement or only partial alleviation of her symptoms. The use of etanercept resulted in remission of aphthous stomatitis and considerable improvement of quality of life. The use of newer biological for selected and severe manifestations of CD may lead to improved morbidity in these patients, but more studies are indicated.

## Competing interests

The authors declare that they have no competing interests.

## Authors’ contributions

AH and HP carried out conception and design of the case report, AH, JL and GY carried out literature review, AH, HP, JL, GY and GK carried out drafting the case report and final approval of the version to be published, all authors read and approved the final manuscript.
